# Clinically significant association of elevated expression of nuclear factor E2-related factor 2 expression with higher glucose uptake and progression of upper urinary tract cancer

**DOI:** 10.1186/s12885-018-4427-1

**Published:** 2018-05-02

**Authors:** Akinori Nukui, Takahiro Narimatsu, Tsunehito Kambara, Hideyuki Abe, Setsu Sakamoto, Ken-Ichiro Yoshida, Takao Kamai

**Affiliations:** 1 Department of Urology, Dokkyo Medical University, 880 Kitakobayashi Mibu, Tochigi, 321-0293 Japan; 2 ET Center, Dokkyo Medical University Hospital, Mibu, Tochigi, Japan

**Keywords:** Upper urinary tract urothelial cancer, Nuclear factor E2-related factor 2 (Nrf2), Positron emission tomography (PET), [^18^F]fluorodeoxyglucose (^18^F-FDG), Maximum standardized uptake value (SUVmax)

## Abstract

**Background:**

There is growing evidence that the transcription factor nuclear factor E2-related factor 2 (Nrf2) is the major participant in regulating antioxidants and pathways for detoxifying reactive oxygen species (ROS), as well as having a vital role in tumor proliferation, invasion, and chemoresistance. It was also recently reported that Nrf2 supports cell proliferation by promoting metabolic activity. Thus, Nrf2 is involved in progression of cancer. Upper urinary tract urothelial carcinoma (UTUC) is a biologically aggressive tumor with high rates of recurrence and progression, resulting in a poor prognosis. However, the role of Nrf2 in UTUC is largely unknown.

**Methods:**

In order to study the role of Nrf2 in UTUC from the metabolic perspective, we retrospectively assessed Nrf2 expression in the surgical specimen and the preoperative maximum standard glucose uptake (SUVmax) on [^18^F]fluorodeoxy-glucose positron emission tomography (^18^F-FDG-PET) of 107 patients with UTUC who underwent radical nephroureterectomy.

**Results:**

Increased expression of Nrf2 in the primary lesion was correlated with less differentiated histology, local invasion, and lymph node metastasis, and was also an independent indicator of shorter overall survival according to multivariate analysis. Furthermore, increased expression of Nrf2 was associated with higher preoperative SUVmax by the primary tumor on ^18^F-FDG-PET, while Nrf2 expression and SUVmax were also significantly correlated in the metastatic lymph nodes. Among the 18 patients with lymph node metastasis at nephroureterectomy who underwent retroperitoneal lymph node dissection and received adjuvant chemotherapy, the patients with higher Nrf2 expression in the primary tumor had worse recurrence-free survival.

**Conclusions:**

These results suggest that constitutive activation of Nrf2 might be linked with tumor aerobic glycolysis and progression of UTUC, indicating that Nrf2 signaling in the tumor microenvironment promotes progression of UTUC.

**Electronic supplementary material:**

The online version of this article (10.1186/s12885-018-4427-1) contains supplementary material, which is available to authorized users.

## Background

Among urothelial tumors, upper urinary tract urothelial carcinoma (UTUC) is relatively uncommon and only accounts for about 5% of such malignancies [1]. The gold standard for treatment of localized UTUC is radical nephroureterectomy with resection of a bladder cuff. However, UTUC is characterized by frequent recurrence and the outcome tends to be poor with progression to advanced or metastatic disease, even after early diagnosis [2–4], probably because occult micrometastases occur at surgery since the ureter is a thin-walled structure with rich lymphatic drainage. Previous pathological studies have demonstrated that poorly differentiated histology, invasion of the muscular layer, metastasis to lymph nodes, and lymphovascular invasion (LVI) are associated with recurrent UTUC and are poor prognostic factors [5–7]. However, none of the current biomarkers fulfill the clinical and statistical criteria for improving detection of this cancer, predicting the outcome, making decisions about treatment, or monitoring the response to therapy. Therefore, novel prognostic molecular markers that can identify the development and progression of UTUC are needed.

Since cancer cells are exposed to an increase in oxidative stress, antioxidants are beneficial to cancer growth. The Kelch-like ECH-associated protein 1 (Keap1)- nuclear factor erythroid-2-related factor 2 (Nrf2) pathway has a major role in protective responses to both oxidative and electrophilic stress. On the other hand, cancer cells depend on aerobic glycolysis to provide energy due to metabolic reprogramming, which is known as the Warburg effect [8]. To develop into a more aggressive phenotype, cancer cells must generate energy for cell proliferation, cell division, evasion of the host immune response, and survive [9]. There are many lines evidence that various metabolic pathways have a profound effect on cancer metabolism in the tumor microenvironment [9, 10]. Recent reports revealed that Nrf2 promotes various metabolic pathways including serine and transketolase, and cell proliferation in cancers [11, 12]. Transketolase has roles in production of antioxidant nicotinamide adenine dinucleotide phosphate (NADPH) to counteract oxidative stresses. Mitsuishi et al. showed that Nrf2 promoted the pentose phosphate pathway, a major biochemical pathway that generates antioxidant NADPH, under the sustained activation of phosphatidylinositol 3‘kinase (PI3K) - serine/threonine kinase Akt signalling, with supporting cell proliferation in addition to enhancing cytoprotection, indicating that activation of Nrf2 promotes the process of metabolic reprogramming triggered by proliferative signals [13]. The PI3K-Akt pathway is perturbed in various cancers, with interruption of this pathway by targeting agents showing antiproliferative, antiviability, antiangiogenic, and proapoptotic effects. In addition, activation of Akt disrupts the transcription of glucose transporter protein-1 (GLUT-1) and its translocation to the plasma membrane, promoting glucose utilization independently of any proliferative effect [14]. Activation of Nrf2 benefits malignant cells by promoting chemoresistance and proliferation, with aberrant elevation of Nrf2 indicating a poor prognosis in various human cancers, including lung, head and neck, breast, ovarian cancers [15–17]. Thus, constitutive Nrf2 activation is important for the development and progression of human cancers.

We hypothesized that activation of Nrf2 might be associated with progression of UTUC from the metabolic perspective. However, no studies have shown the metabolic significance of Nrf2 in human UTUC. Since uptake of [^18^F]fluorodeoxy-glucose (^18^F-FDG) can be used to measure glucose consumption by aerobic and anaerobic glycolysis, ^18^F-FDG-positron emission tomography (^18^F-FDG-PET) was developed to exploit high glucose uptake/utilization by tumor cells, providing a clinically powerful tool for metabolic evaluation of many cancers, and useful for assessing on-target inhibition of the PI3K-Akt pathway [14, 18]. Furthermore, it has been reported that assessment of metabolic activity by ^18^F-FDG-PET might be possible in urothelial cancer [19]. Accordingly, we studied Nrf2 expression in surgically resected tumors and the preoperative maximum standard glucose uptake (SUVmax) on ^18^F-FDG-PET in UTUC patients and investigated the relationship between the expression levels of Nrf2 and the SUVmax in order to assess the roles of Nrf2 in this disease. This may be the first report about the association of Nrf2 with metabolic perspective in UTUC based on SUVmax data determined with ^18^F-FDG-PET, and it provides information about the biological significance of metabolic and antioxidant responses in this cancer.

## Methods

### Patients

We retrospectively studied 107 consecutive Japanese patients, in whom primary UTUC without distant metastasis (cT_any_N_any_M0) was diagnosed from 2007 to 2015. Patients routinely underwent preoperative computed tomography (CT) and/or magnetic resonance imaging (MRI) to acquire staging information. After 2011, ^18^F-FDG-PET/CT was also performed in 42 patients for preoperative staging. Whole-body imaging with a combined ^18^F-FDG PET/CT scanner (Biograph, Sensation 16, Siemens Systems) and data processing were done as reported previously [20, 21]. The pretreatment SUVmax value on ^18^F-FDG PET was defined as the baseline SUVmax. Surgery was performed before any other therapy was given. In addition to nephroureterectomy, lymphadenectomy was added if enlarged lymph nodes (> 0.8 cm in diameter) were confirmed. Lymphadenectomy was performed as follows: medial to the ureter in ureteropelvic tumors, border vena cava or right side of the aorta for right side and border aorta for left side in renal pelvic tumors and/or higher ureteral tumors, and common/external/internal iliac and obturator region in lower ureter tumors [22, 23].

Three specimens of tumor tissue and various specimens of non-neoplastic tissue from the renal pelvis and/or ureter were harvested from each patient during nephroureterectomy and were stored at −80°C, as described previously [7]. Clinical staging was performed according to the TNM classification [24]. This study was conducted according to the Declaration of Helsinki and it was approved by the ethics board of Dokkyo Medical University Hospital. Before nephroureterectomy, a consent form approved by our institutional Committee on Human Rights in Research was signed by each patient.

In 18 patients who had lymph node metastasis at nephroureterectomy, adjuvant systemic chemotherapy with the GC regimen (gemcitabine at 1000 mg/m^2^ on days 1, 8 and 15; and cisplatin at 70 mg/m^2^ on day 2) or the MVAC regimen (methotrexate at 30 mg/m^2^ on days 1, 15 and 22; vinblastine at 3 mg/m^2^ on days 2, 15 and 22; Adriamycin at 30 mg/m^2^ on day 2; and cisplatin at 70 mg/m^2^ on day 2) was given every 4 weeks, starting at 1 to 1.5 months after nephroureterorectomy. Patients generally received 2 or 3 courses of GC or MVAC therapy due to their advanced age and postoperative renal insufficiency.

### Western blotting and immunohistochemistry

Stromal tissue was carefully dissected from the samples of tumor tissue and normal tissue. We performed western blotting with an anti-Nrf2 monoclonal antibody (Abcam, # ab-62,352, Cambridge, UK), as described previously [20]. After protein bands were visualized by chemiluminescence, densitometry was performed with a PDI imaging scanner (Agfa Japan, Tokyo) and data were analyzed by using NIH Image software (ImageJ for Mac OS, version 1.50). Nrf2 expression was calculated relative to beta-actin expression in both the tumor specimens and the corresponding non-tumor specimens. Densitometric analysis was employed for semiquantification of Nrf2 expression, with the relative amount of Nrf2 protein in each tumor specimen being calculated as the ratio of the optical density of the tumor tissue to that of the corresponding non-tumor tissue (set at 1.0) [25].

To confirm the results of western blotting, representative tumor specimens from 15 patients were subjected to immunohistochemistry, employing the same antibodies used for western blotting [26]. Surgical specimens were transferred to 10% buffered formalin and fixed overnight. The fixed samples were embedded in paraffin, and serially sliced into 5-μm sections. After dewaxing, sections were autoclaved at 120 °C for 1 min in 10 mM sodium citrate buffer (pH 6.0) and immersed in 0.3% H_2_O_2_. They were then incubated overnight at 4 °C with primary antibodies against Nrf2 (diluted 1:200, Abcam, # ab-62,352, Cambridge, UK). The sections were rinsed with phosphate buffered saline and then treated with EnVision peroxidase conjugates (Dako, Hamburg, Germany) for 30 min. The sections were then stained with 3.3′-diaminobenzidine tetrahydrochloride and counter-stained with hematoxylin. Nrf2 expression on tumor cell was determined semiquantitatively as described previously [27, 28]: 0, 1+, 2+, and 3+. In each patient, Nrf2 staining was scored by assessing 500 to 1000 cancer cells in 5–10 microscopic fields of 3 to 7 sections. The sections were scored independently by two authors (AN and TK).

### Statistical analysis

The Mann-Whitney *U* test was used to compare western blotting data between two groups (regional lymph node involvement and LVI), while the Kruskal-Wallis test was employed for comparisons among three groups (histological grade) or among four groups (T stage). Associations between immunohistochemical staining intensity for Nrf2 and expression levels of Nrf2 by western blotting were analyzed by the Kruskal-Wallis test, and those between immunostaining for Nrf2 and pathological characteristics were analyzed by Pearson’s χ^2^ test. Spearman’s rank correlation coefficient analysis was performed to investigate the correlation between Nrf2 expression by western blotting and SUVmax. Kaplan-Meier analysis was used to estimate survival, with differences being assessed by the log-rank test. The survival impact of Nrf2 expression, tumor grade, pT stage, regional lymph node metastasis, and LVI was assessed by univariate and multivariate Cox proportional hazards analysis. In all analyses, *P* <  0.05 was considered significant. Analyses were performed using EZR (Saitama Medical center, Jichi Medical University, Japan) [29].

## Results

In this cohort, 107 patients (69 men and 38 women) aged from 51 to 85 years (mean age: 67.3 years) were studied. Postoperative follow-up ranged from 3 months to 91 months (median: 19 months).

### Association of Nrf2 and SUVmax with pathologic characteristics

**Fig. 1:**
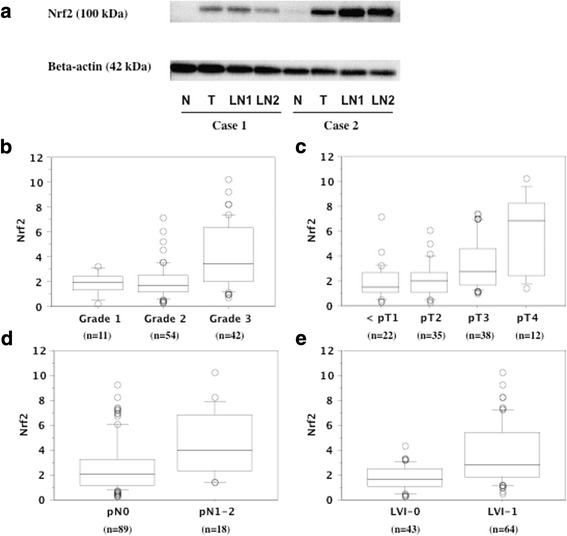
Representative expression of Nrf2 in the primary tumor using Western blotting and its association with pathologic characteristics. **a**. M; marker. N; non-tumor tissue. T; primary tumor tissue. LN; lymph node tissue. LN1 and LN2 were metastatic lymph node. The expressions of Nrf2 in the primary tumors were associated with tumor grade (**b**), pT stage (**c**), regional lymph node metastasis (**d**), and lymphovascular invasion (LVI) (**e**). The median value is the central line, the box is the interquartile range, the bars are the full range, and the points are the outliers

Nrf2 protein was detected in both tumor tissues and non-tumor tissues by western blotting (Fig. 1a), but showed significantly higher expression in tumor tissues (mean ± S.D. = 2.9 ± 2.1) compared with non-tumor tissues (set at 1.0). While the abundance of expression of Nrf2 was not different between the locations of the tumors (renal pelvis vs. ureter), the larger tumors (> median of 2.7 cm in diameter) showed increased expression (Table 1). Elevated expression of Nrf2 in the primary tumor was significantly associated with less differentiated histology (*P* <  0.0001), local invasion (*P* <  0.0001), lymph node metastasis (*P* = 0.0084), and LVI (*P* <  0.0001) (Table 1, Figs. 1b-e).

**Table 1:** Relationship between pathologic characteristics and Nrf2 and SUVmax

	Nrf2			SUVmax		
	Number	mean ± S.D	*p* value	Number	mean ± S.D	p value
tumor	107	2.9 ± 2.1	P < 0.0001	42	7.3 ± 3.6	
non-tumor	107	set at 1.0				
Location						
renal pelvis	48	2.8 ± 1.3	*P* = 0.4729	19	7.8 ± 3.2	*P* = 0.6273
ureter	59	3.3 ± 1.7		23	7.4 ± 3.8	
Size (diameter)						
2.7 cm >	54	2.1 ± 1.5	*P* < 0.0001	21	4.6 ± 2.5	*P* = 0.0029
> 2.7 cm	53	4.8 ± 2.9		21	9.9 ± 5.1	
Grade						
Grade 1	11	2.3 ± 1.2	P < 0.0001	6	4.3 ± 0.3	P = 0.0043
Grade 2	54	2.0 ± 1.4		21	5.8 ± 1.7	
Grade 3	42	3.8 ± 2.5		15	9.4 ± 4.5	
pT						
< pT1	22	1.9 ± 1.4	P < 0.0001	7	5.4 ± 2.2	P = 0.0024
pT2	35	2.1 ± 1.4		13	5.9 ± 1.1	
pT3	38	3.0 ± 1.8		14	8.9 ± 3.8	
pT4	12	5.7 ± 3.2		8	12.5 ± 5.6	
pN						
pN0	89	2.5 ± 1.9	P = 0.0084	29	6.9 ± 3.1	*P* = 0.0162
pN1–2	18	4.2 ± 2.7		13	13.7 ± 5.2	
LVI						
LVI 0	43	1.8 ± 0.9	P < 0.0001	17	5.8 ± 1.9	*P* = 0.0234
LVI 1	64	3.8 ± 2.4		25	8.3 ± 4.3	

**Fig. 2:**
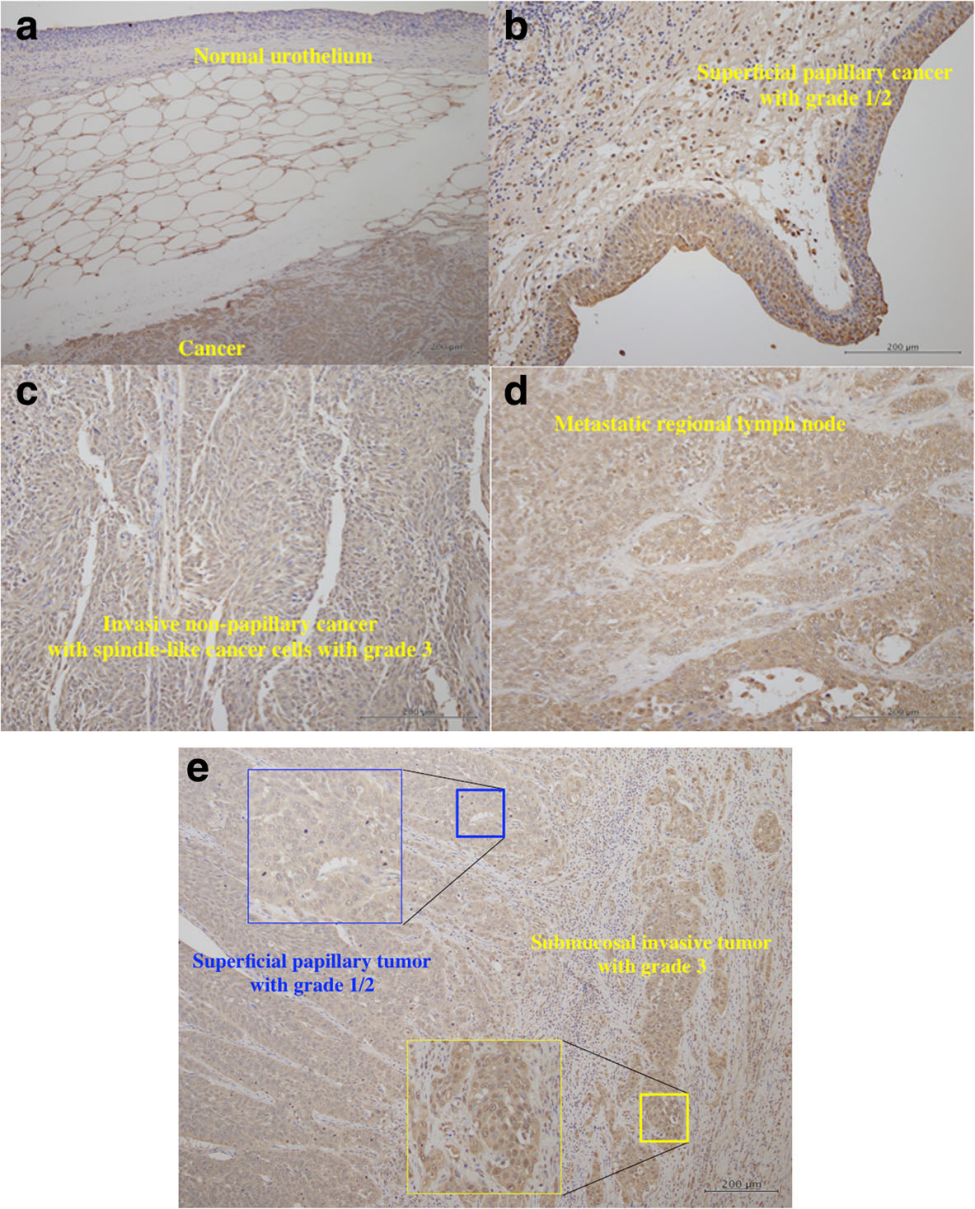
Representative immunohistochemistry in the primary tumor tissues for anti-Nrf2 antibody. Cancer cells showed positive reaction, but normal uroepithelium did not (**a**). Superficial tumors with grade 1/2 showed heterogeneous staining in nuclear and cytoplasm (**b**). Many of tumor cells in invasive tumors with grade 3 showed strong staining in nuclear and cytoplasm (**c**). The tumor cells in metastatic lymph node showed moderate to strong staining in nuclear and cytoplasm (**d**). In a case of invasive pT3 tumor, submucosal invasive tumor cells with grade 3 showed intense staining in nucleus and cytoplasm compared to superficial papillary tumor with grade 1/2 showing weak to moderate staining in cytoplasm with negative to weak nuclear staining (**e**)

In immunohistochemistry, Nrf2 was detectable in nearly all tumor cells, but not the normal urothelium (Fig. 2a). In many of tumor cells, cytoplasmic Nrf2 was expressed, while nuclear Nrf2 expression was heterogeneous. The tumor cells with high grade 3 showed almost homogeneous intense reaction for anti-Nrf2 antibody in both the nucleus and cytoplasm. In contrast, the tumor cells with lower grade 1/2 showed weak to moderate reaction in cytoplasm with heterogeneous nuclear Nrf2 immunostaining (Figs. 2b-e). The tumors with intense Nrf2 immunohistochemical expression showed increased expression of Nrf2 detected by western blotting (Additional file 1 Figure S1A). Higher immunostaining of Nrf2 was associated with poorer differentiation, local invasion, regional lymph node involvement, and LVI (Additional file 1 Figure S1B).

**Fig. 3:**
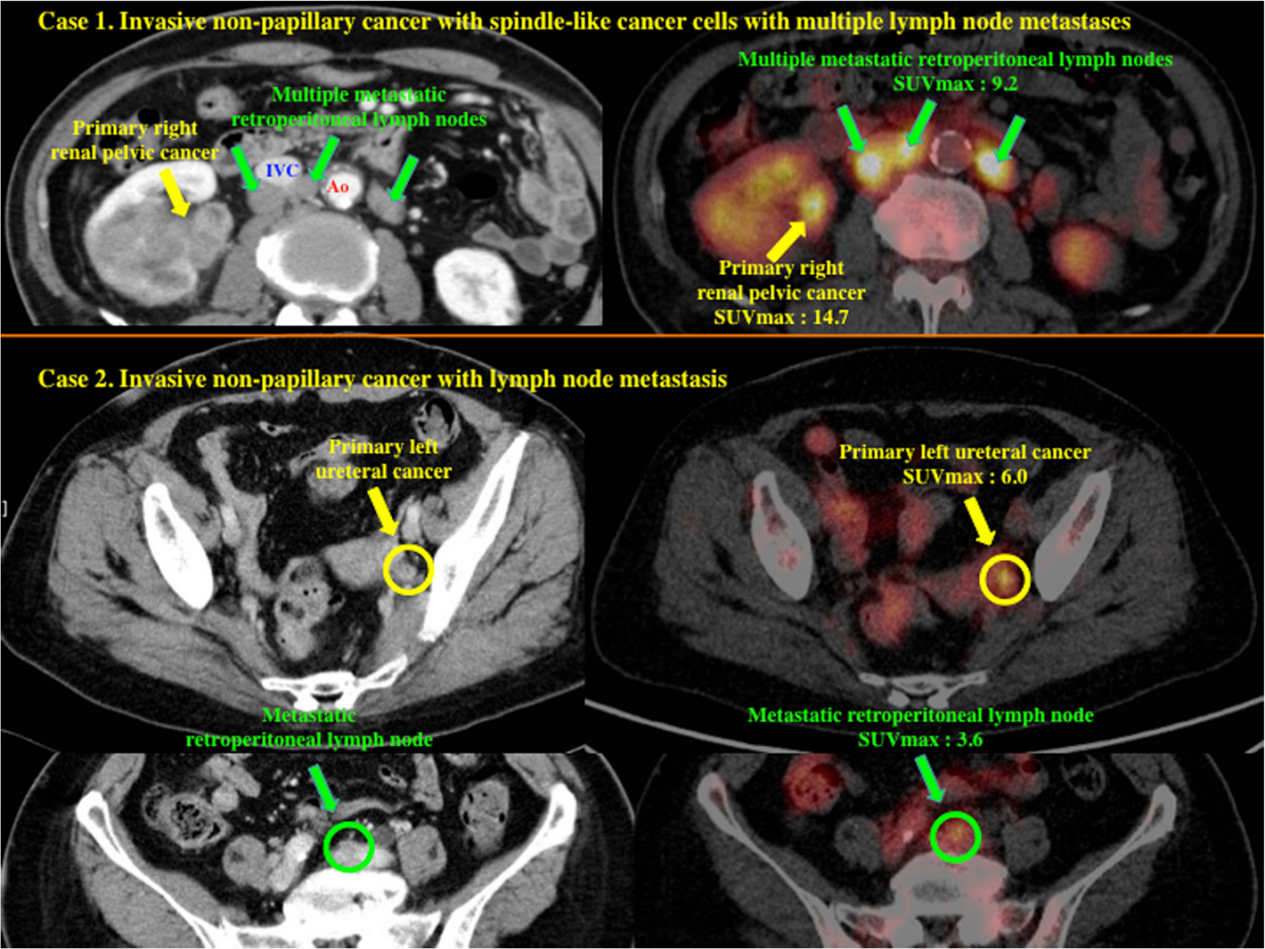
Representative images of PET-CT Case 1 presented with primary right renal pelvic tumors and multiple retroperitoneal lymph nodes involvement. Case 2 presented with primary left ureteral tumors with metastatic retroperitoneal lymph node. Ao; abdominal aorta, IVC; inferior vena cava

**Fig. 4:**
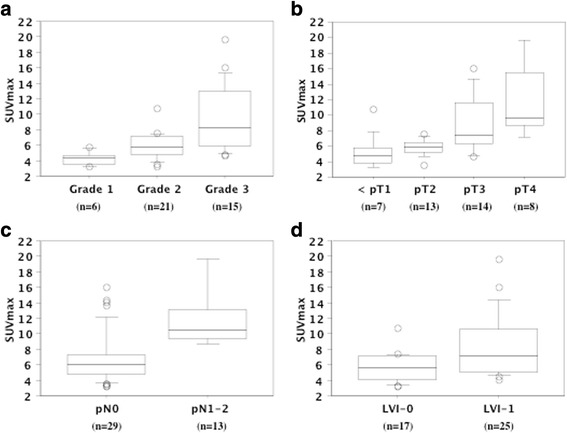
SUVmax in pathologic characteristics. The maximum standard glucose uptake (SUVmax) on^18^F-FDG-PET in the primary tumors was associated with tumor grade (**a**), pT stage (**b**), regional lymph node metastasis (**c**), and lymphovascular invasion (LVI) (**d**). The median value is the central line, the box is the interquartile range, the bars are the full range, and the points are the outliers

In the 42 patients who underwent ^18^F-FDG-PET after 2011, SUVmax could be calculated for all 42 primary tumors (mean ± S.D. = 7.3 ± 3.6) and for 13 metastatic regional lymph nodes of 5 patients (6.8 ± 1.9) (Fig. 3). A higher SUVmax of the primary tumor was significantly related to less differentiated histology (*P* = 0.0043), local invasion (*P* = 0.0024), lymph node metastasis (*P* = 0.0162), and LVI (*P* = 0.0234) (Table 1, Fig. 4).

**Fig. 5:**
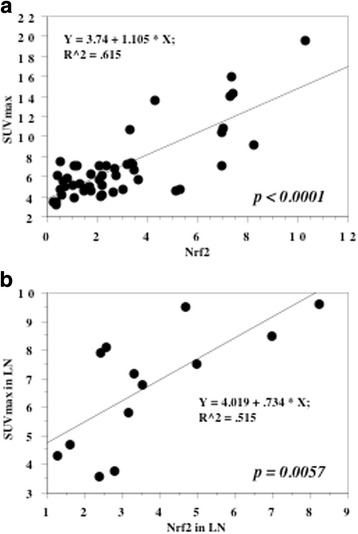
Spearman rank correlation coefficient relationship. X axis is an independent variable. Y axis is a dependent variable. Expression levels of Nrf2 in the primary tumors and in metastatic lymph node were positively associated with SUVmax value in the primary tumors (**a**) and in the metastatic lymph node (**b**), respectively

We analyzed the relation between Nrf2 expression and tumor SUVmax in these 42 patients by using Nrf2 expression as an independent variable and SUVmax as a dependent variable. In the 42 primary tumors, Nrf2 expression and tumor SUVmax showed a significant positive correlation (r^2^ = 0.615, *P* <  0.0001, Fig. 5a). In the 13 lymph nodes with histologically confirmed metastasis, a significant positive correlation was also noted between Nrf2 expression (3.7 ± 2.0) and SUVmax (6.8 ± 1.9) (r^2^ = 0.515, *P* = 0.0057, Fig. 5b).

### Prognostic impact of Nrf2 and SUVmax

The median level of Nrf2 expression in the primary tumors was 2.2, so the patients were divided into a high expression group (*n* = 53) and a low expression group (*n* = 54) at this cut-off value. Similarly, the median SUVmax of the 42 primary tumors investigated in this study was 6.2, and a high SUVmax group (*n* = 21) and a low SUVmax group (n = 21) were formed by dividing the patients at this cut-off value.

**Fig. 6:**
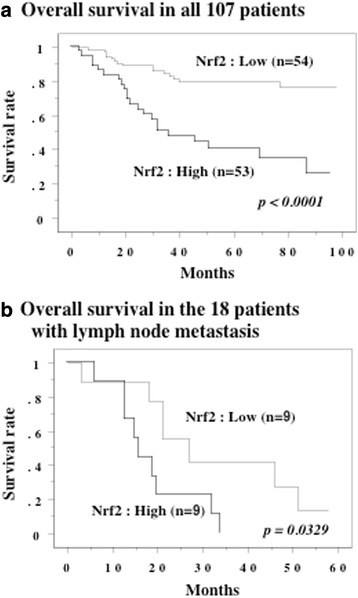
Overall survival curve. The cases were divided into two groups at the median values of Nrf2 in the primary tumors - high and low expression. **a**: All 107 patients. **b**: The 18 patients with lymph node metastasis at the time of nephroureterectomy received adjuvant chemotherapy with the GC or MVAC regimens

According to Kaplan-Meier curves for patients with low or high Nrf2 expression, increased Nrf2 expression in the primary tumor was associated with worse overall survival (*P* <  0.0001, Fig. 6a). A high SUVmax of the primary tumor was also correlated with worse overall survival (*P* <  0.0001). Univariate analysis by the Cox proportional hazards model revealed that tumor grade, pT stage, lymph node metastasis, LVI, and Nrf2 all had a significant influence on survival (Table 2). In addition, multivariate analysis confirmed that pT stage, lymph node metastasis, LVI, and Nrf2 were independent prognostic factors.

**Table 2:** Cox regression analysis for various potential prognostic factors in overall survival

Variable	Unfavorable/ favorable characteristics	No. of Patients	Univariate (U)			Multivariate (M)		
			Relative risk	95% confidential interval	P value	Relative risk	95% confidential interval	*P* value
Grade	3 / 2 / 1	42 / 54 / 11	1.868	1.086–3.211	0.0239	1.047	0.345–1.219	0.1399
pT	4 / 3 / 2 / 1	12 / 38 / 35 / 22	2.762	1.855–4.113	< 0.0001	1.814	1.041–3.160	0.0355
pN	2,1 / 0	18 / 89	3.030	1.985–4.626	< 0.0001	1.912	1.210–3.020	0.0055
lymphovascular invasion (LVI)	1 / 0	64 / 43	11.175	3.981–31.365	< 0.0001	5.451	1.364–21.795	0.0165
Nrf2	high / low	53 / 54	4.063	2.119–7.792	< 0.0001	2.242	1.040–4.832	0.0393

The 18 patients with lymph node metastasis at nephroureterectomy received adjuvant chemotherapy with the GC or MVAC regimens. The median level of Nrf2 expression in the primary tumors of these patients was 4.0, and patients with lower Nrf2 expression had longer recurrence-free survival (*P* = 0.0329, Fig. 6b).

## Discussion

Because tumor cells are exposed to high levels of reactive oxygen species (ROS), minimizing oxidative stress is a requirement for survival and constitutive activation of Nrf2 is important for adaptation to a pro-oxidative environment. In addition to regulation of oxidative stress, a recent investigation into the metabolic role of Nrf2 in proliferating cells showed that it activates the pentose phosphate pathway and diverts metabolites of glucose to de novo nucleotide synthesis [13]. Thus, Nrf2 has a dual role and can promote either cancer prevention or progression depending on the cellular context and microenvironment [15–17]. We investigated the influence of Nrf2 on UTUC metabolism by investigating the relationship between Nrf2 expression and SUVmax in these tumors, demonstrating the following points in this study. First, Nrf2 expression was higher in the tumor tissues than in non-tumor tissues. Second, elevated expression of Nrf2 in the primary tumor was related to less differentiated histology, local invasion, lymph node metastasis, and LVI. Third, we found a positive correlation between Nrf2 expression in tumor tissues and the SUVmax on ^18^F-FDG-PET. Fourth, increased tumor expression of Nrf2 was correlated with worse overall survival and was an independent prognostic factor for survival. We think that this is the first report about the association of Nrf2 with metabolic reprogramming in UTUC based on SUVmax data determined with ^18^F-FDG-PET. Our observations suggested that aberrant elevation of Nrf2 expression occurs in UTUC, and that constitutive activation of this transcription factor is associated with progression of the tumor, biological aggressiveness, and a worse prognosis.

In order to study what cells Nrf2 is expressed in the tissues, immunohistochemistry analysis was performed. The immunohistochemical expression of Nrf2 was increased in nearly all tumor cells compared with non-tumor cells in UTUC. In addition to cytoplasm staining, the tumor cells showed heterogeneous nuclear staining. Much of the tumor cells with nuclear Nrf2 immunostaining showed poorer histological differentiation. These observations might be consistent with the findings obtained in western blotting, in which Nrf2 expression was elevated in the tumor cells with poorer differentiation. In non-small cell lung carcinoma, nuclear Nrf2 expression was highly heterogeneous, and positive nuclear Nrf2 expression was associated with worse recurrence-free survival [27]. In gastric cancer, the Nrf2 protein was detected mainly in the nucleus of tumor cells, and Nrf2 positivity was closely associated with poorer differentiation and poorer overall survival [28]. Since the nuclear Nrf2 stabilization result in its transcriptional activity increases in cancer cells, which contributes to tumor cell chemoresistance and proliferation [15–17], it is likely that the rise of Nrf2 activity mainly occur in more malignant undifferentiated tumor cells [30]. However, we should study the roles of cytoplasmic Nrf2, as well as nuclear Nrf2.

Tumor cells require a constant supply of nutrients to maintain energy metabolism and protein synthesis for rapid proliferation [9]. This elevated demand may be met by increasing nutrient availability through vasculogenesis and/or through enhancement of cellular uptake via the up-regulation of certain transporters [31]. Due to the well-established influence of energy metabolism on the growth and development cancer, its reprogramming is viewed as one of the “hallmarks of cancer” [8]. Switching the metabolism of glucose from mitochondrial oxidation to glycolysis (Warburg effect) is typically employed for generation of adenosine triphosphate by tumor cells [32], and increased uptake of glucose is a key change associated with elevation of glycolysis in cancer cells.

In the present study, a higher SUVmax of the primary tumor was associated with higher histological grade, tumor invasion, lymph node metastasis, LVI, and worse survival, as was also the case for increased Nrf2 expression, indicating that increased glucose uptake and acceleration of glycolysis were related to more aggressive behavior of UTUC. The renal pelvis and ureter are thin-walled structures with rich lymphatic drainage, so local invasion of UTUC and/or lymph node metastasis are common at diagnosis [1]. In UTUC patients, LVI is associated with features of biological aggressiveness and strongly predicts both recurrence and cancer-specific mortality [5–7]. The present study showed that lymph node metastasis, LVI, and Nrf2 expression were all independent prognostic factors for overall survival. We previously reported that patients with lymph node metastasis at nephroureterectomy had LVI, and that LVI-positive patients who were N0 M0 at the time of surgery showed early recurrence with a worse prognosis [7]. Therefore, LVI might be important in progression to lymph node metastasis. In the present study, patients with LVI and lymph node metastasis showed elevation of both Nrf2 expression and SUVmax in the primary tumor, while metastatic lymph nodes showed increased Nrf2 expression and SUVmax values compared with normal lymph nodes. Interestingly, a significant positive correlation between Nrf2 expression and SUVmax was observed for both the primary tumors and the metastatic lymph nodes. Nrf2 is reported to redirect glucose and glutamine toward anabolic pathways, enhancing metabolic activity and growth to facilitate tumor proliferation [13]. Therefore, it is likely that constitutive activation of Nrf2 might be associated with LVI and lymph node metastasis in UTUC.

In the present study, 18 patients with lymph node metastasis at nephroureterectomy received adjuvant chemotherapy (GC or MVAC). Among them, the patients with longer recurrence-free survival had lower Nrf2 expression and lower SUVmax values in both the primary tumors and metastatic lymph nodes compared to the patients with recurrence. There is evidence that cancer cells use the Nrf2 system for adapting to stress in the tumor microenvironment, thus promoting tumor survival [17]. Constitutive activation of Nrf2 signaling has been found in several tumors and cancer cell lines, and is associated with more rapid tumor growth and with resistance to chemotherapy. Nrf2  signaling shows up-regulation after exposure of cancer cells to chemotherapy agents, and this alteration is associated with acquisition of resistance [16, 33]. Thus, our findings suggest that increased Nrf2 expression and elevated aerobic glycolysis might be linked to chemoresistance and enhancement of tumor cell growth.

Our finding that increased expression of Nrf2 in the primary tumor was correlated with elevation of SUVmax, invasive and metastatic tumor behavior, and a worse prognosis suggests that UTUC may show impairment of oxidative phosphorylation with a shift to aerobic glycolysis. However, our study had several limitations, including a retrospective design, investigation of a relatively small number of patients, and a follow-up period too short for definite conclusions to be drawn. The findings of the present study require further validation, preferably by a prospective controlled clinical trial performed on a larger scale. In addition, any mechanistic insight with respect to how Nrf2 increase in cancer cells in UTUC is unclear in this study. Regarding the mechanism underlying activation of Nrf2, it is possible that dysregulation of tumor suppressor genes and oncogenic pathways may be responsible in cancer cells [16, 17]. According to Mitsuishi et al., PI3K-Akt pathway activation augments nuclear accumulation of Nrf2 and supports cell proliferation as well as enhancing cytoprotection [13]. More detailed investigation of the role of the Nrf2 pathway and cross-talk with other signaling systems, such as the PI3K-Akt pathway, may eventually lead to new treatments for UTUC. There have been reports about somatic mutations of Keap-1 and Nrf2 in many human cancers [16]. Tumorigenic mutations of Keap-1 and Nrf2 typically result in activation of Nrf2 targets, indicating the important role of Nrf2 in cancer. Since various single nucleotide polymorphisms (SNPs) might influence the outcome of anticancer therapy, analysis of SNPs for Nrf2, as well as those for Keap1, could also be important to elucidate the effects of the Keap1/Nrf2 pathway in patients with UTUC. To confirm the clinical utility of measuring tumor tissue Nrf2 expression, a prospective study needs to demonstrate its usefulness for assistance in making decisions that improve clinical outcomes.

## Conclusions

We studied the role of Nrf2 in upper urinary tract cancer from the metabolic perspective. We found that increased expression of Nrf2 in the primary tumor was related to local invasion, lymph node metastasis, and elevated ^18^F-FDG uptake, as well as shorter overall survival. Increased expression of Nrf2 was an independent prognostic factor for overall survival. Constitutive activation of Nrf2 might be linked with tumor aerobic glycolysis and progression of upper urinary tract cancer.

## Additional file

Additional file 1 Figure S1 Association between immunostaining intensity for Nrf2 and expression levels of Nrf2 detected by western blotting and pathological characteristics. (A). The tumors with intense immunostaining of Nrf2 showed increased expression of Nrf2. X–axis is intensity of immunestaining of Nrf2. Y-axis for Nrf2 is a ratio of the optical density for the tumor specimen to that for the corresponding non-neoplastic specimen (set at 1.0) by western blotting. The median value is the central line, the box is the interquartile range, the bars are the full range, and the points are the outliers. (B). Higher immunostaining of Nrf2 was associated with poorer histological grading, local invasion (pT), regional lymph node metastasis (pN), and lymphovascular invasion (LVI). (TIFF 1521 kb)

## Abbreviations

^18^F-FDG: [^18^F]fluorodeoxy-glucose
Akt: Protein kinase B
CT: Computed tomography
GC: Gemcitabine and cisplatin
GLUT-1: Glucose transporter protein-1
Keap1: Kelch-like ECH-associated protein 1
LVI: Lymphovascular invasion
MRI: Magnetic resonance imaging
MVAC: Methotrexate, vinblastine, Adriamycin and cisplatin
NADPH: Nicotinamide adenine dinucleotide phosphate
Nrf2: Nuclear factor erythroid-2-related factor 2
PET: Positron emission tomography
PI3K: Phosphatidylinositol 3‘kinase
pT: Pathological Tumor
ROS: Reactive oxygen species
S.D.: Standard deviation
SNPs: Single nucleotide polymorphisms
SUVmax: Maximum standard glucose uptake value (SUVmax)
TNM: The TNM classification of Malignant Tumors (T: tumor, N: lymph node, M: metastasis)
UTUC: Upper urinary tract urothelial carcinoma.
