# Supplementary figures and images for: Clinically significant association of elevated expression of nuclear factor E2-related factor 2 expression with higher glucose uptake and progression of upper urinary tract cancer
Source: BMC Cancer. 2018 May 2;18:493. doi: 10.1186/s12885-018-4427-1 (PMC5930508; doi:10.1186/s12885-018-4427-1)

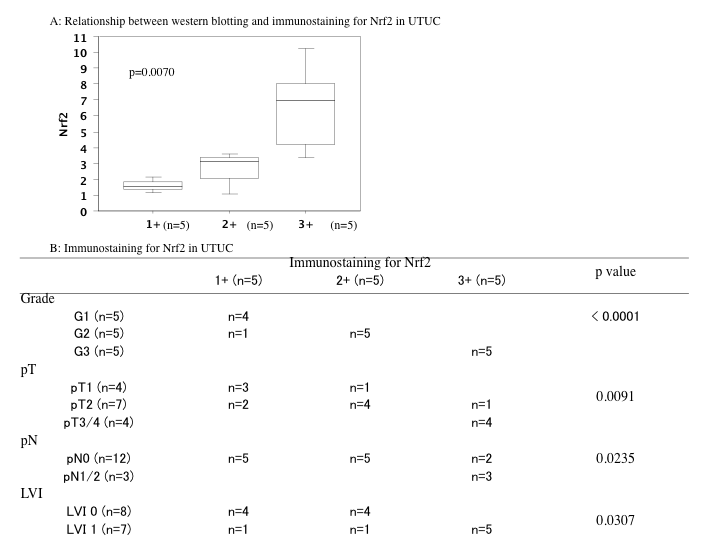

Supplement: Supplementary file 1 — Figure S1 Association between immunostaining intensity for Nrf2 and expression levels of Nrf2 detected by western blotting and pathological characteristics. (A). The tumors with intense immunostaining of Nrf2 showed increased expression of Nrf2. X–axis is intensity of immunestaining of Nrf2. Y-axis for Nrf2 is a ratio of the optical density for the tumor specimen to that for the corresponding non-neoplastic specimen (set at 1.0) by western blotting. The median value is the central line, the box is the interquartile range, the bars are the full range, and the points are the outliers. (B). Higher immunostaining of Nrf2 was associated with poorer histological grading, local invasion (pT), regional lymph node metastasis (pN), and lymphovascular invasion (LVI). (TIFF 1521 kb) [file 12885_2018_4427_MOESM1_ESM.tiff]
